# Effect of Neudesin Neurotrophic Factor on Differentiation of Bovine Preadipocytes and Myoblasts in a Co-Culture System

**DOI:** 10.3390/ani11010034

**Published:** 2020-12-26

**Authors:** Anqi Li, Xiaotong Su, Yaning Wang, Gong Cheng, Linsen Zan, Hongbao Wang

**Affiliations:** 1 College of Animal Science and Technology, Northwest A&F University, Xianyang 712100, China; 13562170853@163.com (A.L.); xiaotongsu1114@163.com (X.S.); wangyn1992@outlook.com (Y.W.); chenggong@nwafu.edu.cn (G.C.); zanlinsen@163.com (L.Z.); 2 National Beef Cattle Improvement Centre, Xianyang 712100, China

**Keywords:** bovine, NENF, preadipocytes, myoblasts, co-culture

## Abstract

**Simple Summary:**

Marbling beef refers to the red and white beef formed by depositing fat between muscle fibers, and “marbling” is an important factor affecting beef quality. Therefore, we established a co-culture system of adipocytes and myocytes to mimic the microenvironment of marbling beef in vivo. However, Neudesin neurotrophic factor (NENF) was not detected in the co-culture system, but was detected in both adipocytes and myoblasts cultured separately. Further studies revealed that *NENF* knockdown inhibits adipogenesis and promotes myogenesis in separately cultured preadipocytes and myoblasts. However, because the monoculture system does not include the interaction of bovine adipocytes and myoblasts in the formation of marbling beef, in this study we investigated the effect of recombinant protein NENF on the differentiation of adipocytes and myoblasts in the co-culture system. The addition of NENF inhibited the formation of lipid droplets in co-cultured adipocytes but had no significant effect on myotube formation. These results were different from, and even conflicted with, those in the monocultures, which suggested that regulation of *NENF* expression in the same cell type changed along with the cell microenvironment and the molecular mechanism of marbling beef formation cannot be fully revealed through studies on the monoculture system.

**Abstract:**

In this study, we successfully established a co-culture system of bovine preadipocytes and myoblasts to explore the effect of exogenous addition of Neudesin neurotrophic factor (NENF) recombinant protein on the differentiation of adipocytes and myoblasts in co-culture. The optimal concentration of NENF recombinant protein was 100 pg/mL. *NENF* promoted the differentiation of bovine preadipocytes and inhibited the differentiation of bovine myoblasts when the cells were cultured separately. After adding NENF recombinant protein to the co-culture system, the accumulation of lipid droplets in bovine preadipocytes decreased, but the differentiation of bovine myoblasts did not change significantly. The results of real-time quantitative PCR (RT-qPCR) and Western blot showed that the expression levels of adipogenesis-related factors such as *PPARγ*, *FABP4* and *FASN* were significantly down-regulated at the mRNA and protein levels in adipocytes, while myogenic marker genes such as *MYOD1*, *MYOG* and *MYHC* had no significant changes at the mRNA or protein levels in myoblasts. This differs from, and potentially conflicts with, the monoculture system, where *NENF* expression in each cell type changed with the cell microenvironment. Consequently, the molecular mechanism of marbling beef formation cannot be fully revealed using monocultures of adipocytes or myocytes.

## 1. Introduction

With the rapid development of the Chinese economy, the consumption of beef is growing rapidly [1,2]. Fat deposits between muscles can reduce muscle density, and after heating, the taste is fresh and succulent [3]. On the cellular level, in marbled beef, adipocytes and myocytes are intermingled, and the two kinds of cells can regulate each other through autocrine and paracrine signaling, and ultimately affect the quality of beef [4,5,6]. Therefore, exploring the formation of intramuscular fat in beef cattle is a major focus of research.

Neudesin neurotrophic factor (NENF), was originally named Neudesin, also known as candidate oncogene *GIG47*, belongs to the membrane-associated progesterone receptor (MAPR) protein family [7,8]. NENF is a multifunctional secretory protein involved in nervous system development, energy metabolism and tumorigenesis. The role of NENF in energy metabolism is mainly focused on fat production and fat metabolism [8,9,10,11]. NENF is a type of secretory protein with neurotrophic activity, which was first found in mouse embryos [12]. NENF can temporarily promote the proliferation of neural progenitor cells in the early stage of development and has a significant effect on neuronal differentiation [13]. The neurotrophic activity of recombinant NENF combined with exogenous heme (NENF-hemin) in primary cultured neurons and Neuro2a cells was significantly higher than that of recombinant NENF, showing that the activity of NENF depends on hemin [14]. In addition, NENF is a important central regulator of food intake [15], and also functions in maintaining the hippocampal anxiety circuit [16].

White adipose tissue (WAT) is closely related to energy metabolism. Studies have found that NENF is highly expressed in mouse WAT, which can play a negative role in the early stage of adipocyte differentiation by regulating the mitogen-activated protein kinase (MAPK) signal pathway [8]. In addition, NENF is closely related to tumorigenesis [7] and is a candidate target for anticancer therapy for many types of cancer cells [17]. At present, research on NENF is mainly focused on humans and model animals, while research on agricultural animals has not been conducted. Research on NENF in the growth and development of bovine myoblasts and the interaction between fat and muscle has not been reported.

The co-culture system is a mixture of two or more kinds of cells, which can better study the influences of the interaction between cells and the effect of two or more kinds of cell proliferation and differentiation under this interaction. In animal bodies, muscle tissue and adipose tissue are developed from mesenchymal tissue of the mesoderm layer. Multiple studies have confirmed an interaction between muscle and adipose tissue from a common precursor [18,19]. The earliest study on the mutual regulation of adipocytes and myocytes was successfully established in sheep in 1997 [20]. In recent years, the co-culture system has been used to study the direct interaction between myocytes and adipocytes [21,22]. Adding trans-10 and cis-12 conjugated linoleic acid to the co-culture system of human preadipocytes and myoblasts increased the expression of adipogenic genes [23]. Choi et al. (2013) found that the co-culture system of bovine preadipocytes and muscle satellite cells increased the expression of adipogenic genes in myoblasts and promoted fat formation [24]. In addition, the expression of IL-6 in muscle cells induced by adipocytes may inhibit differentiation of muscle cells through the autocrine loop [25]. The study of this interaction through the co-culture system can identify important regulatory genes and proteins that affect adipogenesis and skeletal muscle development, which is essential for the preliminary elucidation of the molecular mechanism of marbled beef formation.

NENF is a differentially secreted protein, which was screened and identified by High-Performance Liquid Chromatography/Mass spectrometry (HPLC/MS) in previous research in our laboratory. NENF was secreted in adipocytes and myocytes, but was not detected in a fat-muscle co-culture system. Our previous work revealed that interfering with the *NENF* gene inhibits adipose differentiation and promotes myocyte differentiation in separately cultured cells [26]. However, because the monoculture system does not include the interaction of bovine adipocytes and myoblasts in marbling beef, it cannot reflect the in vivo cellular microenvironment. Therefore, the goal of this study was to determine the function of NENF in the fat-muscle cell co-culture system. We thus co-cultured bovine adipocytes and myoblasts to mimic a marbling beef microenvironment and investigated the effect of recombinant protein NENF on the differentiation of adipocytes and myoblasts, which will be useful for revealing the molecular mechanism of marbling beef formation.

## 2. Materials and Methods

### 2.1. Sample Source

In this study, all animal procedures conformed with the Regulations for the Administration of Affairs Concerning Experimental Animals (Ministry of Science and Technology, China, 2004), and were approved by the Institutional Animal Care and Use Committee (College of Animal Science and Technology, Northwest A&F University, China, No. 2013-23, 20 April 2013).

In this study, preadipocytes and myoblasts was isolated by using the 4-day-old Qinchuan beef cattle which was born and raised at the breeding farm of the National Beef Cattle Improvement Center (Yangling, China).

### 2.2. Isolation and Culture of Bovine Myoblasts and Preadipocytes

For isolation and culture of pre-adipocytes we refer the reader to Meissburger et al. [27]. On this basis, our laboratory established an optimized method for digestion using type I collagenase (Gibco) [28]. We refer the reader to Wang et al. to isolation and culture of myoblasts with Collagenase type Ⅱ (Gibco) and neutral protease (Sigma, Kawasaki City, Japan) [29]. The complete growth medium containing Dulbecco’s Modified Eagle Medium/F-12 (DMEM/F-12, Gibco, Grand Island, NY, USA), 10% fetal bovine serum (FBS, Gibco) and 1% penicillin/streptomycin was used to culture the isolated preadipocytes; the complete growth medium containing 20% FBS was used to culture myoblasts. The cell culture medium was changed every two days. In this experiment, cells differentiated under natural conditions.

### 2.3. Establishment of a Co-Culture System

A 24 × 24 cm^2^ cell cover glass was placed in the middle of a 6 cm Petri dish. Then, a 3.5 cm Petri dish was used to create an isolation ring by making a hole in the bottom of the 3.5 cm Petri dish and placing it upside down in the 6 cm Petri dish. The cover glass was then isolated from the surrounding environment, and single-cell cultures (preadipocytes or myoblasts) were initiated on the cover glass; co-cultures (preadipocytes and myoblasts) were initiated outside the isolation ring. After the cells adhered to the wall, the 3.5 cm dishes were removed, and the co-culture system of bovine adipocytes and myoblasts was successfully established. Complete growth medium containing 10% FBS was used to Co-culture system.

### 2.4. Detection of Differentiation of Preadipocytes and Myoblasts

The NENF recombinant protein (R&D systems, Emeryville, CA, USA) was added to bovine preadipocytes and myoblasts. According to existing research [8,12,13,14], four different final concentration gradients (10 pg/mL, 100 pg/mL, 1 ng/mL and 10 ng/mL) were set for the recombinant NENF protein, and 10 ng/mL phosphate-buffered saline (PBS) was added to the control group. When the cells were at 80% confluency, the different concentrations of recombinant protein NENF were added, which was recorded as day 0. At this time, the cells were able to differentiate without induction. The cells were observed continuously for 6 days, and NENF recombinant proteins were added every two days. The optimal concentration was selected by observing the formation of lipid droplets and myotubes on the 2nd, 4th and 6th days (D2, D4 and D6).

The adipocytes to be treated were washed three times with PBS, fixed with 4% paraformaldehyde for 30 min, then washed three times with PBS. Subsequently, the adipocytes were stained with BODIPY dye (Invitrogen, working solution 1 mg/mL) for 30 min and then washed three times with PBS, stained with 4′,6-diamidino-2-phenylindole (DAPI) (Gibco, Grand Island, NY, USA) for 10 min, and then washed three times with PBS. Finally, the number and size of lipid droplets were observed using an Olympus IX71 microscope (OLYMPUS, Dalian, China), and immunofluorescence images were captured. The differentiation of myocytes was judged by observing the number and size of myotubes using bright field microscopy.

### 2.5. Real-Time Quantitative PCR (RT-qPCR)

The total RNA of myocytes and adipocytes in co-culture system was extracted by Trizol reagent (Takara, Mountain View, CA, USA) and reverse transcribed by Prime Script RT reagent kit (Takara, Mountain View, CA, USA).

In order to normalize the target gene mRNA levels in this study, the internal control in adipocytes used *β-Actin* and the internal control in myoblasts used glyceraldehyde-3-phosphate dehydrogenase (*GAPDH*). Subsequently, the cDNA was used for RT-qPCR in triplicate wells by the SYBR Green Real-Time PCR Master Mix (Takara, Mountain View, CA, USA) in a Bio-Rad Real-Time PCR System (Bio-Rad, Hercules, CA, USA).

The relative gene expression was calculated using $RQ=2^{-\left[\left(Ct1-Ct2\right)-\left(Ct3-Ct4\right)\right]}$. The sequence information of all primers used for qRT-PCR detection in this experiment is shown in Table 1.

### 2.6. Western Blot Analysis

The proteins on days 2, 4 and 6 of adipocyte and myocyte differentiation was extracted by the protein extraction kit (Solarbio, Beijing, China), and the protein expression of the corresponding marker genes for fat differentiation and muscle differentiation were detected. The bicinchonininc acid method (BCA, Takara) was used to determine the protein concentration. Proteins were added into protein sample buffer at a proportion of 4:1, and denatured at 100 °C for 10 min. Polyacrylamide gels at 5% and 12% were prepared, and 20 μg of protein samples were loaded per well. Gels were electrophoresed at 80 V for 30 min, followed by 120 V until the samples ran to the bottom of the gel. The protein samples in the gel were transferred to a polyvinylidene fluoride (PVDF) membrane (Merck Millipore, Burlington, MA, USA), then sealed for 20 min with QuickBlock Western blocking solution (Beyotime Biotechnology, Shanghai, China). GAPDH (rabbit anti-GAPDH, 1:10,000 Abcam, Cambridge, UK, NP_001029206.1), FABP4 (rabbit anti- FABP4, 1:1000, Abcam, NT, UK, NP_776739.1), PPARγ (rabbit anti-PPARγ, 1:1000, Boster, Wuhan, China, NP_851367.1), FASN (rabbit anti-FASN, 1:2000, Abcam, NT, UK, NP_777087.1), MYOD1 (mouse anti-MYOD1, 1:1000, Abcam, NT, UK, NP_001035568.2), MYOG (rabbit anti-MYOG, 1:1000, Abcam, NT, UK, NP_001104795.1) and MYHC (mouse anti-MYHC, 1:200, Abcam, NT, UK) antibodies were added and incubated overnight at 4 °C. Then, the corresponding HRP-conjugated Goat Anti-Rabbit IgG (1:5,000, Sangon Biotech, Shanghai, China) or m-IgGκ BP-HRP (1:1,000, sc-516102, Santa Cruz Biotechnology, Inc, Santa Cruz, CA, USA) were added and incubated at room temperature for 1 h. Finally, the prepared Chemiluminescent HRP Substrate (Millipore, Burlington, MA, USA) was added to the chemiluminescence (CL) of the GelDoc gel XR+ imaging system (Bio-Rad, Hercules, CA, USA).

## 3. Results

### 3.1. Determination of the Optimal Concentration of NENF Recombinant Protein

**Figure 1:**
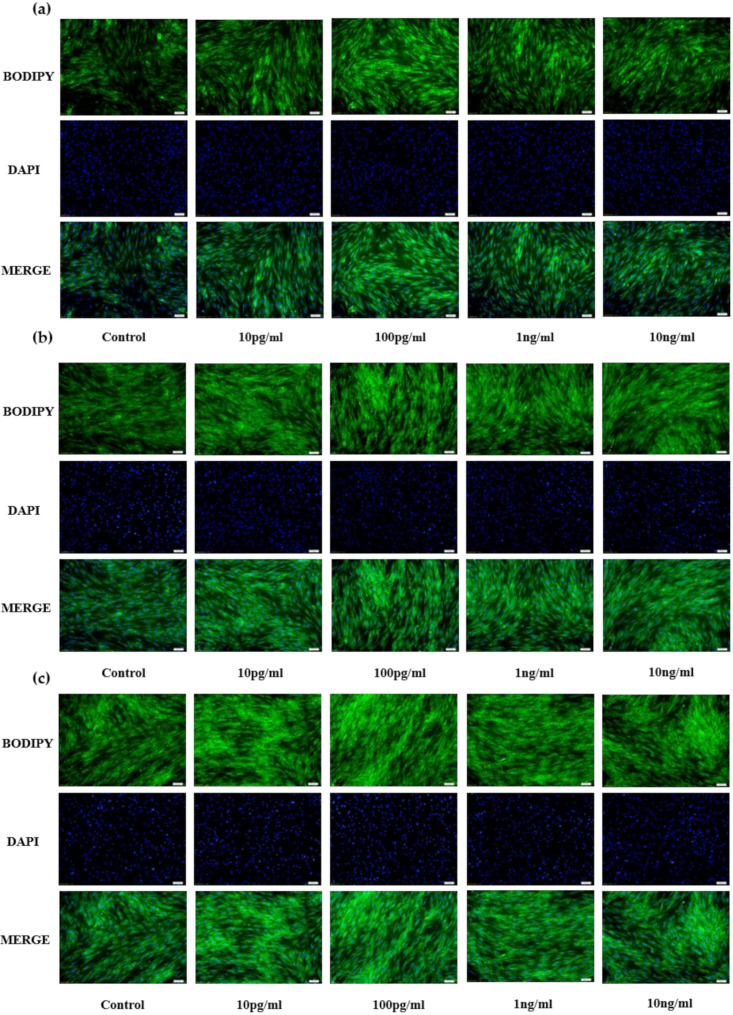
Effects of NENF with different concentrations of preadipocyte differentiation. (**a**) Bovine adipocytes are stained with BODIPY (green) and DAPI (blue) after adding the recombinant protein NENF and PBS at 2nd day of natural differentiation (D2); (**b**) 4th day of natural differentiation (D4); (**c**) 6th day of natural differentiation (D6). (OLYMPUS IX71 100×).

Results for bovine preadipocytes are shown in Figure 1a(D2),b(D4),c(D6). Compared with the control group (PBS), the different concentrations of NENF recombinant protein significantly promoted the accumulation of lipid droplets, and affected the phenotypic differences of three different NENF concentrations at three differentiation stages (D2, D4 and D6). When the concentration of NENF was 100 pg/mL, the accumulation of lipid droplets in bovine preadipocytes was more obvious.

**Figure 2:**
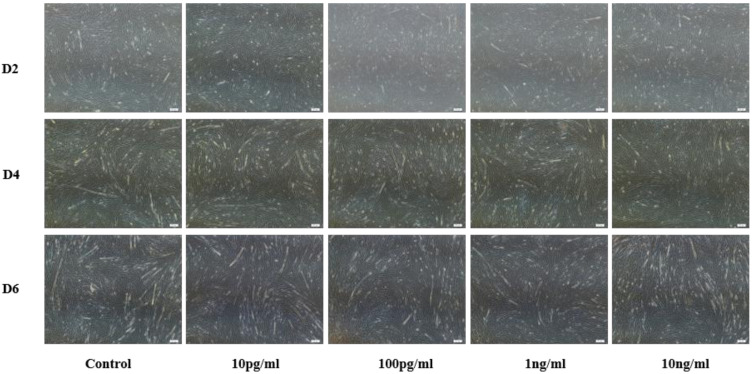
Effects of NENF with different concentrations of myoblast differentiation. Morphological changes of differentiating bovine skeletal primary myoblasts after adding the recombinant protein NENF and PBS at the 2nd day of natural differentiation (D2), 4th day of natural differentiation (D4) and the 6th day of natural differentiation (D6). (OLYMPUS IX71 40×).

Bovine myoblast results are shown in Figure 2. Compared with the control group (PBS), different concentrations of NENF significantly inhibited the formation of myotubes. There were no significant phenotypic differences among the three differentiation stages (D2, D4 and D6). Considering the optimal concentration of preadipocytes, we chose the final concentration of 100 pg/mL NENF.

### 3.2. Exogenous Addition of NENF Recombinant Protein Inhibits the Differentiation of Preadipocytes in Co-Culture System

**Figure 3:**
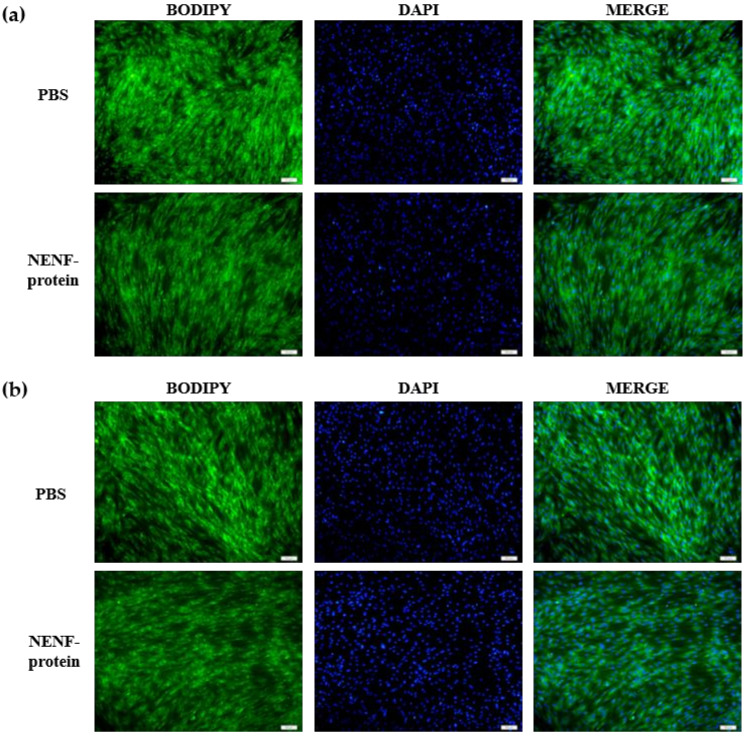
Effects of adding NENF recombinant protein on preadipocyte differentiation in co-culture system. (**a**) Bovine adipocytes are stained with BODIPY (green) and DAPI (blue) after adding the recombinant protein NENF and PBS at 4th day of natural differentiation (D4); (**b**) 6th day of natural differentiation (D6). (OLYMPUS IX71 100×).

**Figure 4:**
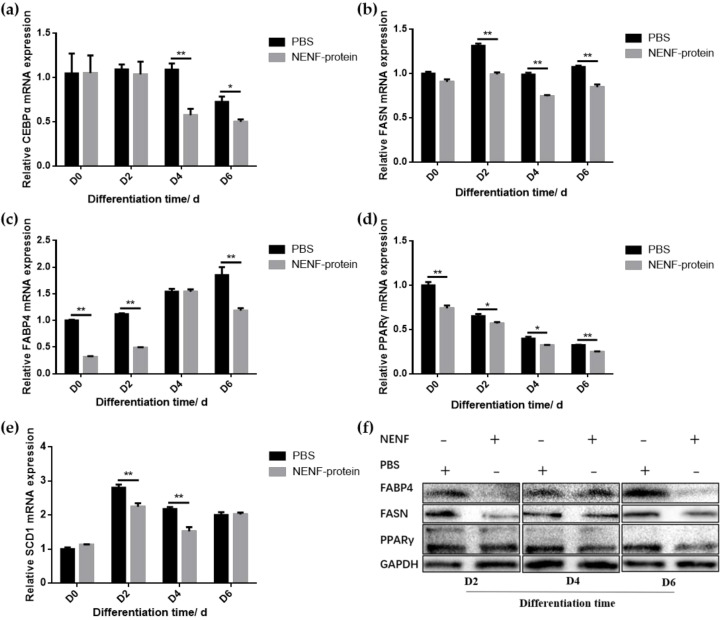
Detection of the expression of adipocyte differentiation marker gene at the mRNA and protein levels. (**a–e**) The expression of lipid-related factors *CEBPα*, *FASN*, *FABP4*, *PPARγ* and *SCD1* at the mRNA level after adding the recombinant protein NENF and PBS at 0th day of natural differentiation (D0), 2nd day of natural differentiation (D2), 4th day of natural differentiation (D4) and 6th day of natural differentiation (D6); (**f**) Western blot results of FABP4, FASN and PPARγ protein expression levels after adding the recombinant protein NENF and PBS at 2nd day of natural differentiation (D2), 4th day of natural differentiation (D4) and 6th day of natural differentiation (D6). Error bars represent s.e.m. * *p* < 0.05; ** *p* < 0.01.

When the adipocytes were at 80% confluence, exogenous NENF recombinant protein was added to the co-culture system. After 4 and 6 days of self-differentiation, the cells were collected for BODIPY staining (Figure 3a,b). Compared with the PBS control group, the NENF recombinant protein treatment group significantly reduced the accumulation of lipids. Then, the expression levels of adipogenic marker genes *CEBPα* (CCAAT enhancer binding protein α), *FASN* (fatty acid synthase), *FABP4* (fatty acid binding protein 4), *PPARγ* (peroxisome proliferator-activated receptors γ) and *SCD1* (stearoyl-CoA desaturase 1) were detected. RT-qPCR analysis showed that exogenous addition of NENF down-regulated the expression of *CEBPα, FASN, FABP4, PPARγ* and *SCD1* mRNA (Figure 4a–e). Western blot analysis results showed that exogenous addition of NENF recombinant protein down-regulated the expression of PPARγ, FABP4, and FASN proteins (Figure 4f). In summary, the exogenous addition of NENF recombinant protein significantly inhibited the differentiation of preadipocytes in the co-culture system.

### 3.3. Exogenous Addition of NENF Had no Significant Effect on the Differentiation of Myoblasts in Co-Culture System.

**Figure 5:**
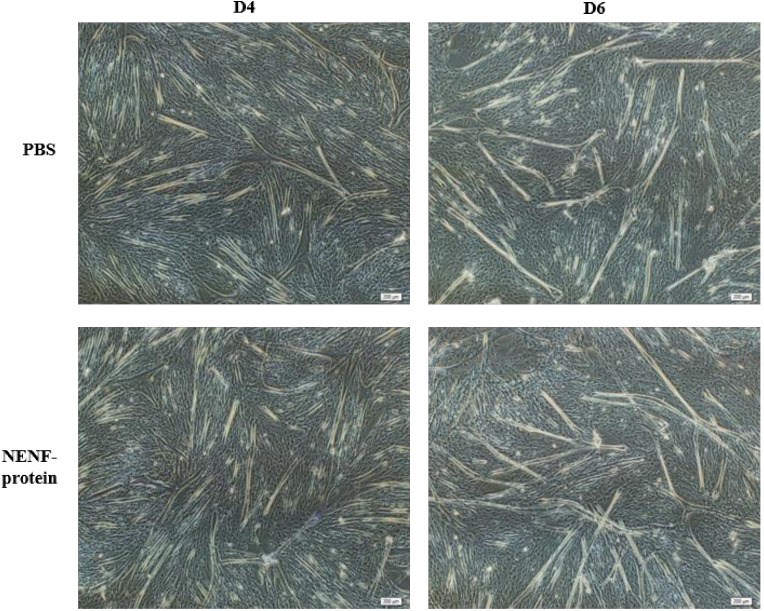
Effects of adding NENF recombinant protein on myoblast differentiation in co-culture system. Morphological changes of myoblasts after adding the recombinant protein NENF and PBS at 4th day of natural differentiation (D4) and 6th day of natural differentiation (D6). (OLYMPUS IX71 40×).

**Figure 6:**
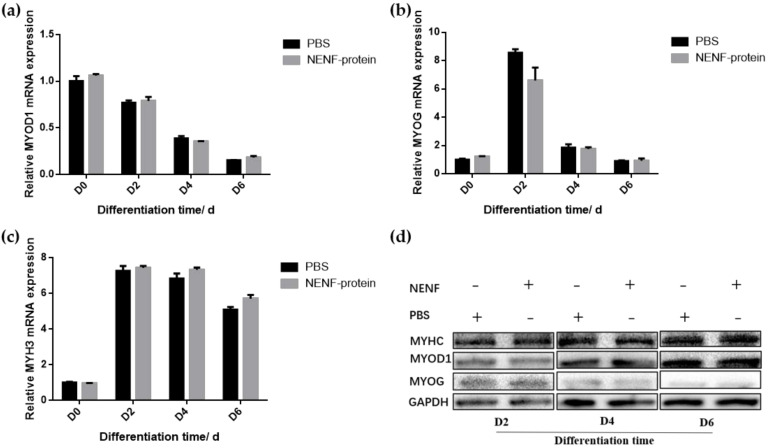
Detection of the expression of myoblast differentiation marker gene expression at the mRNA and protein levels. (**a**–**c**) Relative mRNA expression of *MYOD1*, *MYOG* and *MYH3* after adding the recombinant protein NENF and PBS at 0th day of natural differentiation (D0), 2nd day of natural differentiation (D2), 4th day of natural differentiation (D4) and 6th day of natural differentiation (D6); (**d**) Western blot results of MYOD1, MYOG and MYHC protein expression levels after adding the recombinant protein NENF and PBS at 2nd day of natural differentiation (D2), 4th day of natural differentiation (D4) and 6th day of natural differentiation (D6). Error bars represent s.e.m.

In bovine myoblasts, after 4 and 6 days of self-differentiation, the cell morphology was observed (Figure 5). There was no significant difference in myotube formation between the NENF treatment group and PBS control group. Next, the expression of myogenic marker genes *MYOD1* (myogenic differentiation 1), *MYOG* (myoglobin) and *MYH3* (Myosin-3) was detected. RT-qPCR analysis showed that there were no significant differences in the expression of *MYOD1*, *MYOG* and *MYH3* mRNA due to exogenous NENF recombinant protein compared with the control group (Figure 6a–c). The results of Western blot showed no differences in the expression levels of MYOD1, MYOG and MYHC (myosin heavy chain) at the protein level (Figure 6d). Therefore, the exogenous addition of NENF recombinant protein has no significant effect on the differentiation of myoblasts in the co-culture system.

## 4. Discussion

At present, at the cellular level, most studies on bovine lipid metabolism and muscle development are carried out in separately cultured bovine myocytes or adipocytes [30,31,32,33]. This research method ignores the interaction between myocytes and adipocytes in vivo, and the function and expression regulation of candidate genes for meat quality traits cannot accurately represent the real physiological process. In addition, it cannot be used to evaluate the molecular mechanism of the formation of marbled beef traits. Moreover, myocytes and adipocytes are in the same in vivo growth environment, which is quite different from the cells cultured alone in vitro, so it is not possible to make conclusions from monocultures. Therefore, establishing a co-culture system of bovine preadipocytes and myoblasts in vitro allows researchers to more closely mimic the physiological environment in vivo, and explore the regulation of specific genes on bovine lipid metabolism and muscle development.

In a previous study, we screened and identified the differentially secreted protein NENF, using HPLC/MS. NENF was secreted in both adipocytes and myoblasts, but not in the fat-muscle co-culture system. Moreover, there was no difference in *NENF* at the transcriptional level, which may be due to the inhibition of post-transcriptional expression of the gene in the co-culture system [20]. Previous studies have shown that NENF is directly related to lipogenesis and lipid metabolism. Exogenous Neudesin-hemin can inhibit adipogenesis of 3T3-L1 cells induced by MDI (0.25 µM dexamethasone, 10 µg/mL insulin and 0.5 Mm 3-isobutyl-1-methylxantine). RNAi experiments showed that inhibiting the expression of endogenous NENF can significantly promote adipogenesis [8]. In the monoculture systems, however, we found that interfering with *NENF* promoted the differentiation of bovine myoblasts and inhibited the differentiation of preadipocytes [26], which is contrary to the above results, and may be due to differences in adipocytes among different species. However, the growth environment of the cells themselves is complex, and cells cultured alone cannot reflect the three-dimensional state of cells in the body [15,19,20,34]. In contrast, co-culture systems can be more similar to the environment of the cells in vivo.

In this study, the effect of recombinant NENF protein on the differentiation of bovine preadipocytes and myoblasts was investigated by adding recombinant protein into a fat-muscle co-culture system. First, according to previous research [8,12,13,14,15], the exogenous addition amount of secreted protein NENF was set to 10 pg/mL, 100 pg/mL, 1 ng/mL or 10 ng/mL. Through phenotypic observation, the differences between different concentrations was not significant, especially in myocytes, but the differences between the control group and the treatment groups were significant. Considering that NENF is a kind of secretory protein, the concentration in the culture medium will not be very high, so combined with the experimental results, 100 pg/mL was selected as the final concentration.

Second, exogenous NENF recombinant protein was added to the fat-muscle co-culture system. The quantitative results of BODIPY staining and marker gene mRNA and protein levels showed that the exogenous addition of NENF recombinant protein inhibited the differentiation of preadipocytes, but there was no significant difference in muscle cell differentiation in the co-culture system. This result is not consistent with the function of *NENF* in separately cultured adipocytes and myocytes, and we speculate that this is because the co-culture system is different from the monoculture, and the single cell type culture systems cannot reflect the complex environment and influencing factors in vivo. In the co-culture system, adipocytes and myocytes regulate each other’s proliferation and differentiation through autocrine or paracrine signaling, and can better mimic the real in vivo environment. The secreted protein NENF was not detected in the adipocytes-myoblasts co-culture system, and the co-culture system itself can promote adipogenesis [24]. Therefore, it is speculated that NENF has a negative role in regulating lipid production in the co-culture system and even in the body.

In this study, exogenous addition of NENF did not change the differentiation state of myoblasts in the co-culture system. It is possible that a different dosage of the secreted protein NENF has no significant effect on myogenesis in a co-culture environment or even in vivo. There are relatively few published studies on fat-muscle co-culture in agricultural animals, and thus there are few results that can be used for reference, and *NENF*’s involvement in myocyte differentiation has rarely been reported. From the results of this study, there may be significant differences, or even conflicting results, in the regulation of gene expression between a co-culture system and monocultures, so the specific mechanism needs to be determined by more in-depth study.

## 5. Conclusions

Our study showed that exogenous NENF significantly inhibited the differentiation of bovine preadipocytes in the co-culture system but had no significant effect on the differentiation of bovine myoblasts. The results show that the regulation mode of gene expression in co-culture system is different from that in separately cultured cells, which is worthy of further study.

## Figures and Tables

**Table 1:** Sequence information of the primers of qRT-PCR related genes in this study.

Gene Name	Accession Numbers	Primer Sequence (5′–3′)	Fragments Size (bp)
*β-actin*	NM_173979	Forward: TCTAGGCGGACTGTTAGC	82
		Reverse: CCATGCCAATCTCATCTCG	
*GAPDH*	NM_001034034	Forward: AGTTCAACGGCACAGTCAAGG	124
		Reverse: ACCACATACTCAGCACCAGCA	
*PPARγ*	NM_181024	Forward: TGAAGAGCCTTCCAACTCCC	117
		Reverse: GTCCTCCGGAAGAAACCCTTG	
*CEBPα*	NM_176784	Forward: ATCTGCGAACACGAGACG	73
		Reverse: CCAGGAACTCGTCGTTGAA	
*SCD1*	NM_173959	Forward: TCCGACCTAAGAGCCGAGAA	200
		Reverse: TGGGCAGCACTATTCACCAG	
*FASN*	NM_001012669	Forward: GGCAAACGGAAAAACGGTGA	183
		Reverse: CTTGGTATTCCGGGTCCGAG	
*FABP4*	NM_174314	Forward: TGAGATTTCCTTCAAATTGGG	101
		Reverse: CTTGTACCAGAGCACCTTCATC	
*MYOD1*	NM_001040478	Forward: AACCCCAACCCGATTTACC	196
		Reverse: CACAACAGTTCCTTCGCCTCT	
*MYOG*	NM_001111325	Forward: GGCGTGTAAGGTGTGTAAG	85
		Reverse: CTTCTTGAGTCTGCGCTTCT	
*MYH3*	NM_001101835.1	Forward: AAATGAGGGATGACCGCCTG	205

## Data Availability

No new data were created or analyzed in this study. Data sharing is not applicable to this article.
